# Quality-by-design ecofriendly potentiometric sensor for rapid monitoring of hydroxychloroquine purity in the presence of toxic impurities

**DOI:** 10.1038/s41598-024-53456-8

**Published:** 2024-03-22

**Authors:** Mohammed E. Draz, Fadwa H. Edrees, Heba M. Mohamed, Sherif F. Hammad, Ahmed S. Saad

**Affiliations:** 1 Department of Pharmaceutical Chemistry, Faculty of Pharmacy, Delta University for Science and Technology, Gamasa, Egypt; 2 Pharmaceutical Chemistry Department, Faculty of Pharmacy, Nahda University (NUB), Beni-Suef, 62511 Egypt; 3 Analytical Chemistry Department, Faculty of Pharmacy, Cairo University, Kasr El-Aini St, Cairo, 11562 Egypt; 4 Pharmaceutical Chemistry Department, Faculty of Pharmacy, Helwan University, Helwan, Egypt; 5 Medicinal Chemistry Department, PharmD Program, Egypt-Japan University of Science and Technology (E-JUST), New Borg El-Arab City, Alexandria 21934 Egypt

**Keywords:** Hydroxychloroquine, Quality-by-design, Design of experiment, Potentiometric sensor, Purity testing, Ion-selective electrode, Chemistry, Analytical chemistry, Electrochemistry, Medicinal chemistry

## Abstract

Hydroxychloroquine (HCQ) is prescribed to treat malaria and certain autoimmune diseases. Recent studies questioned its efficiency in relieving COVID-19 symptoms and improving clinical outcomes. This work presents a quality-by-design approach to develop, optimize, and validate a potentiometric sensor for the selective analysis of HCQ in the presence of its toxic impurities (key starting materials), namely 4,7-Dichloroquinoline (DCQ) and hydroxynovaldiamine (HND). The study employed a custom experimental design of 16 sensors with different ion exchangers, plasticizers, and ionophores. We observed the Nernstian slopes, correlation coefficients, quantification limit, response time, and selectivity coefficient for DCQ and HND. The computer software constructed a prediction model for each response. The predicted responses strongly correlate to the experimental ones, indicating model fitness. The optimized sensor achieved 93.8% desirability. It proved a slope of 30.57 mV/decade, a correlation coefficient of 0.9931, a quantification limit of 1.07 × 10^–6^ M, a detection limit of 2.18 × 10^–7^ M, and a fast response of 6.5 s within the pH range of 2.5–8.5. The sensor was successfully used to determine HCQ purity in its raw material. The sensor represents a potential tool for rapid, sensitive, and selective monitoring of HCQ purity during industrial production from its starting materials.

## Introduction

In 1946, researchers introduced hydroxychloroquine (HCQ) as a chloroquine (CQ) derivative by incorporating a hydroxyl group. The purpose was to decrease the ocular toxicity often associated with CQ. It was found that HCQ is approximately three times less toxic compared to CQ^1^. HCQ, shown in Supplementary Figure S1, is a weak base that concentrates in the parasite's acid vesicles to inhibit the polymerization of heme. It inhibits certain enzymes by interacting with DNA^2^. HCQ and CQ are commonly prescribed in treating autoimmune diseases like rheumatoid arthritis, systemic lupus erythematosus, and dermatomyositis. Although HCQ has numerous immunomodulatory effects, its precise mechanism in each condition is unknown^2^. It prevents lysosomal acidification and interferes with proteolysis, chemotaxis, phagocytosis, and antigen presentation^2,3^. Lately, during the COVID-19 pandemic, some studies have introduced CQ or HCQ as possible treatments^4^. A recent study showed that the antimalarial drugs HCQ and CQ significantly affected viral clearance and clinical outcomes compared to the control group^5^. However, the mechanism of action of HCQ against covid 19 virus is still under investigation. It is believed to change the pH of endosomes to prevent virus entry, transportation, and post-entry events. The report suggests halting virus replication and modification and inhibiting glycosyltransferases^6,7^. HCQ and CQ showed several toxicity issues, such as retinopathy, cardiomyopathy, respiratory failure, and others, so the cases should be monitored closely^2^. The industrial production of the HCQ as an active pharmaceutical ingredient (API) reports various toxic impurities (key starting materials) like 4,7-dichloroquinoline (DCQ) and 2-amino-5-diethylaminopentane also known as hydroxynovaldiamine (HND)^8^.

A validated analytical sensor that selectively determines the API in the presence of its key starting materials is an inevitable tool in pharmaceutical plants. It enables chemists to instantaneously monitor the API formation reaction, study the reaction kinetics, and optimize the reaction conditions for maximum yield. Later, the sensor can represent a tool in quality control laboratories to assess API purity. It guarantees an efficient production process and a safe final product.

Literature reports potentiometric sensors to assess HCQ using modified carbon paste electrodes^9^ and coated graphite electrodes^10^ within HCQ dosage forms and in plasma and urine samples^11^. The literature revealed voltammetric methods for HCQ assay. The differential pulse voltammetry method employed a glassy carbon electrode^12^, and square wave voltammetry used a boron-doped diamond and cork graphite electrode to determine HCQ in tablets and urine^13,14^. A cyclic voltammetry method used a β-cyclodextrin Au-nanoparticles electrode to discriminate the R and S isomers of HCQ^15^. Voltammetric methods determined HCQ in the presence of other drugs within their pharmaceutical formulations and biological matrices^16–21^ and in environmental samples^14^. Liquid chromatography was used in some trials to determine HCQ in the presence of impurities and forced degradation products^22,23^.

Yet, there is no reported potentiometric method to determine HCQ in the presence of its main toxic impurities with the intrinsic advantages of portability, convenience for untrained users, and efficiency.

Traditional experiments adopt the trial and error or the one-factor-at-a-time methods. Unfortunately, the two ways are inefficient, unstructured, and unlikely to find the optimum set of conditions across multiple variables. A more effective and efficient experimentation approach is using statistically designed experiments (DOE). It is a systematic, efficient method that enables scientists to rapidly study the relationship between multiple input variables (factors) and critical output variables (responses). By manipulating various inputs simultaneously, DOE can identify crucial interactions that may be missed when experimenting with one factor at a time. All possible combinations can be investigated (full factorial) or only a subset (fractional factorial). It also makes it easier to understand the effects of multidimensional input factors and their interactions on the output responses of specific analytical methods^24,25^. Our research group utilized the paramount benefit of quality by design (QBD) to fabricate an industrial potentiometric sensor for at-line determination of the active pharmaceutical ingredient, HCQ, in the presence of its major toxic synthesis impurities, DCQ and HND. We used a computational approach to optimize the membrane recipe and sensor performance.

## Materials and methods

### Reagents and materials

Standard HCQ, DCQ, and HND were kindly gifted from EVA Pharmaceuticals. All of the chemicals and solvents used were of analytical grade. 2-nitrophenyl octyl ether (NPOE), high molecular weight polyvinyl chloride (PVC), β-Cyclodextrin (BCD), and calix[8]arene (CX8) were provided from Fluka (Steinheim, Germany). Tetrahydrofuran (THF) was purchased from BDH (Poole, England). Dibutyl phthalate (DBP), tetraphenylborate (TPB), and tungstophosphoric acid hydrate (PT) were purchased from (Merck, Darmstadt, Germany). Bi-distilled water was used throughout the work.

### Standard solutions

Stock standard solution of 1 × 10^–2^ M HCQ was prepared in bi-distilled water. Working HCQ solutions (1 × 10^–7^–1 × 10^–2^ M) were done by serial diluting the stock standard solution with bi-distilled water.

Hydrochloric acid and sodium hydroxide solutions were prepared and used to adjust the pH of the medium.

### Instrument

A Jenway digital potentiometer model 3510 (Essex, U.K.) performed the potential measurements with a double junction Ag/AgCl reference electrode, Orion, Thermo Scientific no. 900200.

Custom experimental design and data analysis calculations were done using the Design expert® software version 13.0.1.0 Copyright© 2022, Stat-Ease, Inc, USA.

### Sensors preparation

The experimental design included 16 membrane recipes with varied cation-exchangers (TPB and PT), ionophores (BCD and CX8), and plasticizers (DBP and NPOE), Table 1. Quantitatively, all membranes included a fixed proportion of PVC (32% w/w), cation exchanger (1% w/w), ionophore (2% w/w), and plasticizer (65% w/w). The cocktails were prepared in a 5-ml volumetric flask, using THF as a solvent. The polished surface of the glassy carbon electrode (GCE) was electrochemically coated with polyaniline^26^. Subsequently, 60 µL of each cocktail was individually applied to the modified dry GCE surface and allowed to dry to prepare the sensor. The sensor was then immersed in a 1 × 10^–2^ M HCQ standard solution for one hour before analysis.

**Table 1:** Experimental design showing the studied factors and monitored responses for each sensor.

Run	Factors			Responses					
	A	B	C	Slope (mV/decade)	r	pLOQ (M)	${K}_{HCQ, DCQ}^{Pot}$	${K}_{HCQ, HND}^{Pot}$	Response time (sec)
1	TPB (+)	BCD (+)	DBP (−)	29.49	0.996	5.97	1.50 × 10^–5^	8.27 × 10^–4^	7
2	TPB (+)	BCD (+)	NPOE (+)	22.51	0.984	5.97	6.08 × 10^–5^	1.01 × 10^–4^	12
3	PT (−)	CX8 (−)	NPOE (+)	23.53	0.998	5.97	3.13 × 10^–5^	3.85 × 10^–4^	6
4	TPB (+)	CX8 (−)	DBP (−)	23.53	0.999	5.97	6.28 × 10^–4^	1.86 × 10^–3^	8
5	PT (−)	CX8 (−)	DBP (−)	33.14	0.988	5.97	6.39 × 10^–5^	1.90 × 10^–3^	5
6	PT (−)	BCD (+)	NPOE (+)	23.92	0.999	5.97	2.80 × 10^–4^	9.46 × 10^–4^	5
7	PT (−)	CX8 (−)	DBP (−)	35.32	0.977	5.97	9.39 × 10^–5^	8.59 × 10^–4^	6
8	TPB (+)	CX8 (−)	DBP (−)	23.52	0.997	5.97	2.68 × 10^–4^	2.01 × 10^–3^	6
9	PT (−)	BCD (+)	NPOE (+)	24.47	0.998	5.97	3.69 × 10^–4^	3.08 × 10^–4^	6
10	TPB (+)	BCD (+)	DBP (−)	30.87	0.989	5.97	1.19 × 10^–5^	8.30 × 10^–4^	6
11	TPB (+)	BCD (+)	NPOE (+)	24.43	0.995	5.97	8.15 × 10^–5^	3.50 × 10^–4^	10
12	PT (−)	CX8 (−)	NPOE (+)	22.65	0.997	5.97	1.14 × 10^–5^	4.60 × 10^–4^	5
13	PT (−)	BCD (+)	DBP (−)	32.13	0.997	5.97	6.23 × 10^–4^	1.72 × 10^–4^	6
14	TPB (+)	CX8 (−)	NPOE (+)	28.65	0.995	5.97	7.10 × 10^–4^	1.18 × 10^–4^	8
15	TPB (+)	CX8 (−)	NPOE (+)	29.77	0.993	5.97	1.45 × 10^–4^	6.88 × 10^–4^	10
16	PT (−)	BCD (+)	DBP (−)	30.33	0.995	5.97	9.26 × 10^–4^	3.65 × 10^–4^	7

A: Ion-exchanger, B: Ionophore, C: Plasticizer, r:correlation coefficient, pLOQ = -log the quantification limit, ${K}_{HCQ, DCQ}^{Pot}$: selectivity coefficients for 4,7-dichloroquinoline, ${K}_{HCQ, HND}^{Pot}$: selectivity coefficients for hydroxynovaldiamine.

### Sensors optimization

A custom experimental design determined the best recipe for better sensor characteristics, and the design expert software interpreted the results. The ion exchanger type (TPB and PT), plasticizer type (NPOE–DBP), and ionophore type (BCD and CX8) were selected as independent variables, Table 1. Sixteen experiments were designed, and the sensors were randomly prepared and tested. Calibration graphs were created, and regression equations were generated. The slope (S), correlation coefficients (r), limit of quantification (LOQ), selectivity towards DCQ and HND, and response time were selected as dependent variables. Coefficients and P values were statistically calculated, and the desirability function was used to achieve the final optimization. We set the function settings to achieve the target Nernstian slope of 29.58 mV decade^−1^, maximum correlation coefficient, minimum quantification limit, selectivity coefficient, and response time. Prediction formulae have been generated for all main effects and their interactions, and the desirability function concluded the optimum membrane recipe.

### Construction of calibration curves

The potential difference readings developed between the different sensors and the Ag/AgCl double-junction reference electrode immersed in 25 mL aliquots of HCQ working solutions in the 1 × 10^–7^–1 × 10^–2^ M range were recorded within ± 1 mV. Calibration graphs were plotted between the measured potential difference and the logarithm function of the HCQ molar concentration. Regression equations were computed, and the Nernstian slope, range, and linearity were deduced.

### Validation of the sensor performance

Sensor performance parameters (linearity, LOD, LOQ, selectivity, dynamic response time, response reversibility) were calculated according to IUPAC^27^ recommendations, and validation parameters were calculated according to ICH guidelines^28^.

### Effect of pH

The effect of pH on the sensor performance was examined using 0.1 M HCl and 0.1 M NaOH solutions to alter the pH values of a 1 × 10^−2^ and 1 × 10^−3^ M HCQ solution at room temperature. A potential versus pH curve was plotted.

### Sensors selectivity study

The selectivity of the fabricated sensors was determined using the separate solution method^29^. The selectivity coefficients were calculated for the commonly present interfering ions and the two key starting materials (impurities), DCQ and HND, using the following Eq. ^25^:

1 $$ {\text{Log}}\;\;K_{HCQ. Int}^{Pot} = \frac{{E_{Int} - E_{HCQ} }}{S} + \left( {1 + \frac{{Z_{HCQ} }}{{Z_{Int} }}} \right) \log a_{HCQ} $$

where ${K}_{HCQ, Int}^{Pot}$ is the selectivity coefficient, E is the measured potential, S is the slope, Z is the ionic charge of the interfering ions (Int) and HCQ, and α is the activity.

### Molecular docking study

The 3D SDF files for the studied ionophores (CX8 and BCD), besides the ligand HCQ were downloaded from the PubChem website^30^. The Autdock 4 program and PyMOL molecular graphics system plugin visualizer were used to prepare the host and guest molecules and docking process. The Autodock 4 software examines the interaction between a ligand and host molecules by employing the semiempirical free energy force field and the Lamarckian genetic algorithm to evaluate the binding free energy (docking score). PyMOL was used to screen the binding site virtually and to examine the docking runs^31^. The host and guest molecules were protonated and charged at pH 7. Subsequently, their energies were minimized to select the least energetic conformer, and the final optimized structures were saved for the docking process. The optimized conformers were loaded into the AutoDock 4 program, and the best ten poses were selected from 100 poses for further analysis. The ionophore was kept rigid throughout the docking process, while HCQ exhibited flexibility within the ionophore centers. Subsequently, the PyMOL visualizer analyzed the best poses and rated them based on their binding free energy (docking score).

### Dynamic response time

The dynamic response time was assessed over the concentration range 1.0 × 10^–6^–1.0 × 10^–3^ M. We plotted the potential difference against time to conclude the speed and stability of the sensor response. The time elapsed from sensor immersion to equilibration (± 1.0 mV) was recorded as the sensor response time.

### Sensor lifetime

Sensor calibration was performed using different HCQ concentrations; regression equations were computed to evaluate the initial sensor slope (mV/decade). The procedure was repeated weekly to monitor the change in the slope till it deviated by more than one mV/decade from the initially recorded slope. After use, the sensor was stored in a 1 × 10^–2^ M HCQ solution in a refrigerator.

### Assay of HCQ in Plaquenil® tablets

Five Plaquenil® tablets were weighed to determine the average tablet weight. The tablets were crushed and grinded to a uniform powder. A mass of the powder was accurately weighed and transferred to a 25-mL volumetric flask, and the volume was completed using bi-distilled water to prepare a solution equivalent to 1 × 10^–3^ M HCQ. The solution was 100-fold diluted in a 25-mL volumetric flask using bi-distilled. The potential developed between the optimized sensor and an Ag/AgCl reference electrode was recorded in the former solution. The potential was measured again after the addition of 1 mL 1 × 10^–2^ M HCQ (C_s_ = 1 × 10^–2^ M) standard solution, the change in potential after the addition of the standard solution (ΔE) was recorded, and HCQ concentration (C_HCQ_) was calculated from the standard addition Eq. ^32^:

2 $${C}_{HCQ}={\text{Cs}}\left(\frac{{\text{Vs}}}{{\text{Vx}}+{\text{Vs}}}\right){\left[{10}^{n(\Delta E/S)}-\frac{{\text{Vx}}}{{\text{Vs}}+{\text{Vx}}}\right]}^{-1}$$

where V_x_ and Vs are the volumes of the sample and standard solutions, respectively, while S is the sensor slope in mV per decade.

## Results and discussion

The paramount characteristics of potentiometric sensors, including low cost, experimental simplicity, sensitivity, selectivity, rapid responses, portability, and miniaturization options, have made them an attractive solution for diverse analysis fields^33,34^. Still, the optimum membrane formula is the crucial factor influencing the sensor performance. The PVC matrix is widely used due to its unique features that allow high sensitivity and rapid responses. The membrane cocktail ingredients and the working pH, such as the ion exchanger type, the plasticizer, and the ionophore within the PVC matrix^35,36^, were studied. These factors highly contribute to the sensor performance and efficiency in detecting HCQ in the presence of its impurities. The experimental design evaluates the main effect of different variables and their interaction using the least number of experiments^37^. The design examined combinations of varying ion exchangers (TPB and PT), ionophores (BCD and CX8), and plasticizers (DBP and NOPE) through 16 sensor recipes. The sensors' analytical performance was practically evaluated through their slope, correlation coefficient, quantification limit, selectivity coeffecients towards DCQ and HND, and response time. The experimental design and the measured responses are shown in Table 1.

The hydrophobic nature of the ion exchanger plays a crucial role in regulating the selective extraction of the analyte of interest compared to other available interfering ions; moreover, it allows better solubility in the PVC matrix. The ionic sites enable selective responses and decrease membrane resistance and interference from ions in the same sample solution; the current study compared two cation exchangers, TPB and PT. The plasticizer affects the mobility of the constituents within the membrane, which in turn affects the membrane resistance. It facilitates the formation of ion pairs, thus influencing the slope and response time. They also affect the membrane polarity and control the movement of the membrane components into the external aqueous media^29^; the study utilized two plasticizers of different polarities, DBP and NPOE.

Ionophores are the host molecules with a lipophilic nature and particular polar sites within the hydrophobic pocket used for ionic recognition. The presence of ionophores in the membrane recipe highly affects the selectivity pattern due to the stable complex formed between the ion of interest (HCQ) and the ionophore. Various factors govern the selectivity of ionophore-based membranes, including the capacity of the ionophore to selectively extract the ion of interest and the ions' free energy of transfer across the membrane. The study investigated the effects and interactions of two ionophores, BCD and CX8, on the sensor performance parameters.

### Sensor optimization

Based on the literature surveying, the ion exchangers (TPB-PT), plasticizers (NPOE-DBP), and ionophores (BCD and CX8) were used to fabricate HCQ sensors with the ability to reproduce near-Nernstian slopes while achieving acceptable sensitivity, linearity, and selectivity. Subsequently, a custom experimental design was created and implemented to evaluate each membrane component's main effects and interaction terms on the HCQ sensor performance. Sixteen experimentally designed sensors were fabricated, and their responses were measured and presented in Table 1. The prediction models were constructed for each response, and the coefficients for each main effect and interaction terms were generated, Table 2. The coefficients defined the magnitude and direction of each term on the corresponding response. The *P*-values indicate the significance of each term based on significance level α = 0.05, Table 2. Hence, the computed coefficients and *P*-values were carefully examined to investigate and predict the studied factors' effect and interactions on the selected responses.

**Table 2:** Coefficients and *P*-values of the prediction models.

	Intercept	A	B	C	AB	AC	BC	ABC
Slope	27.39	0.795	0.1225	2.4	0.35125	2.14375	− 1.03625	2.0625
*P*-value		0.0128	0.6364	< 0.0001	0.1966	< 0.0001	0.0032	< 0.0001
r	0.99	8.75 × 10^–5^	− 0.00062	− 0.00136	− 0.00301	− 0.00314		
*P*-value		0.9438	0.6217	0.2888	0.0324	0.0267		
pLOQ	5.97	− 2.22 × 10^–16^	− 2.22 × 10^–16^	− 2.22 × 10^–16^	2.22 × 10^–16^	2.22 × 10^–16^	2.22 × 10^–16^	− 2.22 × 10^–16^
*P*-value		0.3466	0.3466	0.3466	0.3466	0.3466	0.3466	0.3466
${K}_{HCQ, DCQ}^{pot}$	2.7 × 10^–4^	2.98 × 10^–5^	− 2.59 × 10^–5^		− 2.2 × 10^–4^			
*P*-value		0.5639	0.6154		0.0008			
Response time	7.06	− 1.3125	− 0.3125	− 0.6875	0.0625	0.9375	0.1875	
*P*-value		0.0012	0.2987	0.0382	0.8304	0.0091	0.5247	
${K}_{HCQ, HND}^{pot}$	7.61 × 10^–4^	− 8.68 × 10^–5^	2.74 × 10^–4^	3.42 × 10^–4^		− 1.9 × 10^–4^	2.81 × 10^–4^	
*P*-value		0.3082	0.0069	0.0017		0.0389	0.0060	

Factors: A = Ion-exchanger, B = Ionophore, and C = Plasticizer.

The underlined values represent the coefficients and *P*-values of the statistically significant factors.

**Figure 1:**
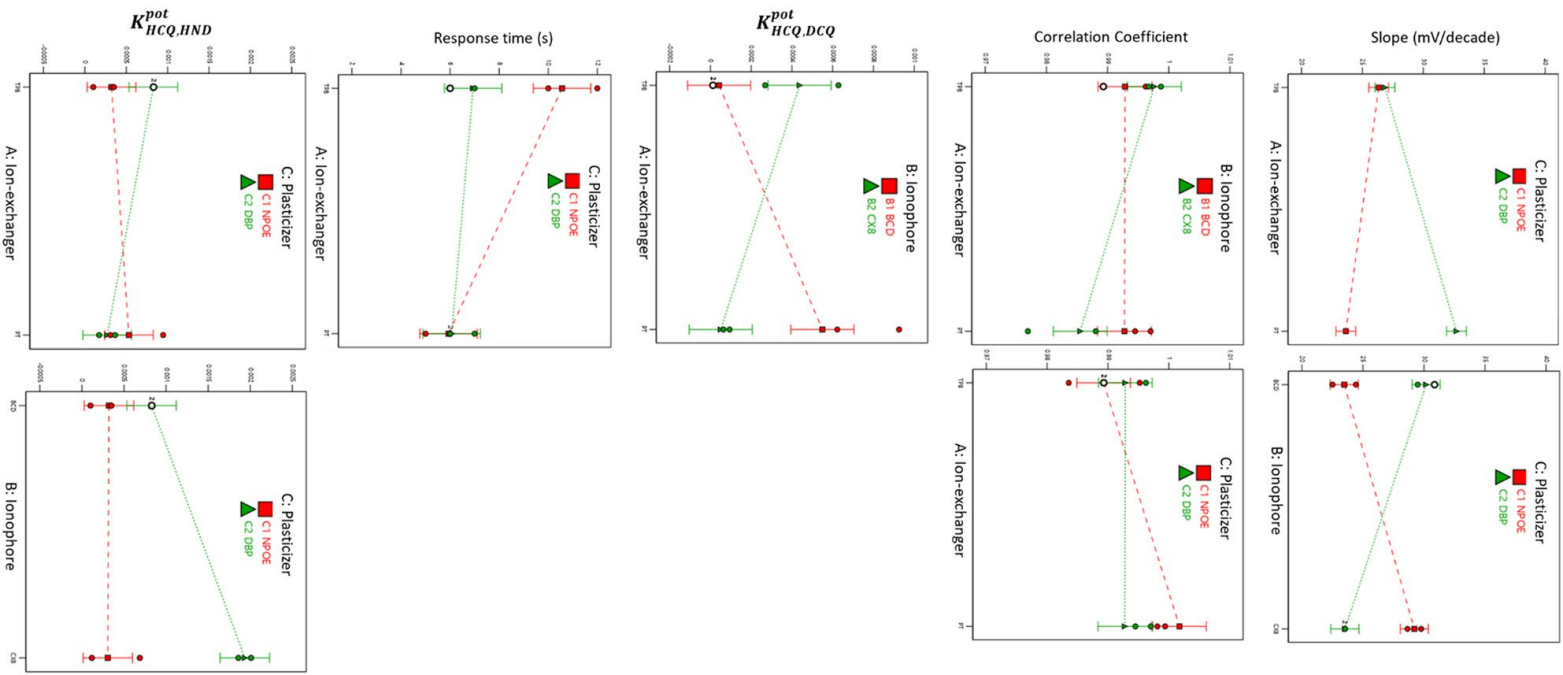
Interaction plots for the studied responses.

The ion exchanger and plasticizer types affected the sensor's slope and response time. On the other hand, the ionophore and plasticizer impact the HND selectivity coefficient (*P*-value < 0.05). All the levels of the studied factors shared the same quantification limit over the 16 sensors, Table 2. However, the ion-exchanger-ionophore interaction influenced the linearity (correlation coefficient) and dichloroquinoline selectivity coefficients. The third interaction term significantly affected the slope, Table 2 and Fig. 1. During optimization, it was crucial to keenly select the level for each statistically significant factor to reach the desired sensor response.

**Figure 2:**
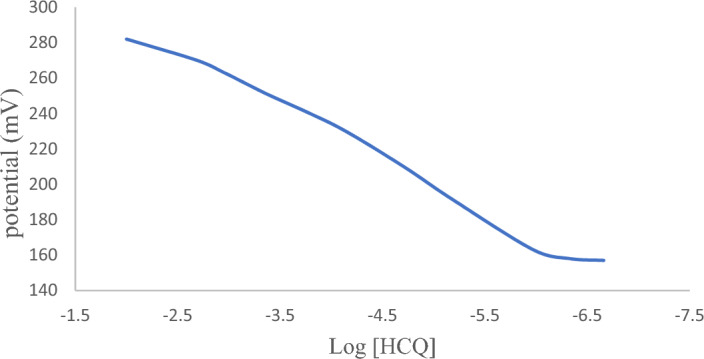
Potential profile of HCQ using the optimized sensor.

Predicting the optimum sensor composition becomes sophisticated in the presence of many responses. The desirability function is an easy, simple, and precise tool for optimization. The optimum membrane components were determined using the response optimizer tool, Supplementary Figure S2. The optimum sensor recipe included TPB as an ion exchanger, BCD as an ionophore, and DBP as a plasticizer, Supplementary Figure S2. The optimized sensor achieved 93.8% desirability, and theoretical responses were practically verified in Fig. 2 and Table 3.

**Table 3:** Electrochemical response characteristics of the optimized sensor.

Parameter		Proposed sensor
Concentration range		1.07 × 10^–6^–1.00 × 10^–2^ M
Linearity	Slope	30.57 mV/decade
	Intercept	351.57 mV
	Correlation coefficient	0.9931
Accuracy (mean ± S.D)^a^		98.65 ± 0.98
Precision (RSD %)	Repeatability^b^	± 1.21
	Intermediate precision^c^	± 1.54
LOD (M)^d^		2.18 × 10^–7^ M
Response time (s)		6.5 ± 1
Working pH range		2.50–8.50
Lifetime (weeks)		6

^a^An average of five determinations.

^b^%RSD for the recovery of three different concentrations, repeated thrice within the same day. (1.11 × 10^–3^, 1.76 × 10^–4^, 1.97 × 10^–5^ M).

^c^%RSD for the recovery of three different concentrations repeated thrice on three successive days.

^d^Limit of detection calculated according to the IUPAC recommendations.

**Figure 3:**
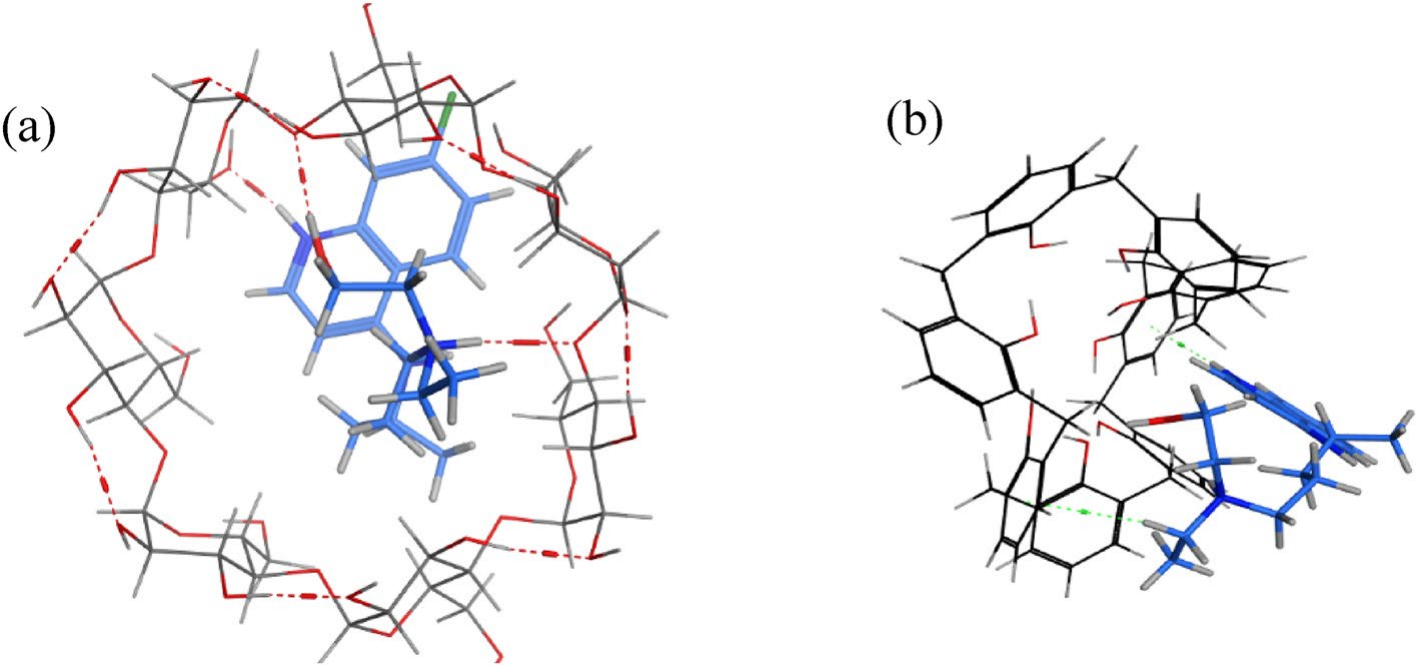
Three-dimensional structures of (**a**) the hydrophobic interaction of β-cyclodextrin with HCQ and (**b**) the hydrophobic interaction of calix[8]arene with HCQ. HCQ appeared as thick blue sticks surrounded by an ionophore represented by thin sticks. The dotted red lines in (**a**) show the H-bonding, and the dotted green lines in (**b**) represent the H-pi bond interactions.

The molecular docking study confirmed the obtained results. The guest molecule (HCQ) achieves a more stable complex with the larger cavity of the BCD host molecule by forming three intermolecular H-bonds, resulting in lower free binding energy $(-5.825 {\text{kcal}}/{\text{mol}})$. On the other hand, HCQ interacts within the relatively small cavity of CX8 through two weaker H-pi intermolecular forces with a higher free binding energy of $-5.271 {\text{kcal}}/{\text{mol}}.$ The docking results show the suitability of the hydrophobic pocket of BCD with HCQ structure, Fig. 3.

### Effect of pH

HCQ is a basic compound. It occurs as a divalent cation in the pH range (2.0–8.5. Upon studying the pH effect through the pH range (1.5–12.5) using two different HCQ concentrations (1 × 10^–2^ and 1 × 10^–3^ M). The potential sharply decreases beyond the pH range of 2.5–8.5 due to the formation of the monoprotonated and deprotonated structures and the proton competition for the active sites in the membrane. The sensor showed stable responses within the pH range of 2.5–8.5, as shown in Supplementary Figure S3.

### Sensor selectivity

The sensor was challenged to selectively determine HCQ in the presence of the common key starting materials (DCQ and HND) and the commonly encountered interfering ions. The potentiometric selectivity coefficients were calculated using the separate solution method, and the results showed that the sensor has higher selectivity for HCQ relative to other interferants, as shown in Supplementary Table S4.

### Method validation

The ICH validation parameters, such as accuracy, repeatability, intermediate precision, linearity, and LOD, were computed, as tabulated in Table 3. We assessed the linearity by plotting the sensor response against the logarithm of HCQ molar concentration. The regression equation elucidated a Nernstian slope with a close-to-unity regression coefficient. The method accuracy was proven through the assay of three different HCQ concentrations (1.11 × 10^–3^, 1.76 × 10^–4^, 1.97 × 10^–5^ M) in thrice. The method repeatability and intermediate precision were evaluated over different HCQ concentrations (1.11 × 10^–3^, 1.76 × 10^–4^, 1.97 × 10^–5^ M) thrice on the same day and through three consecutive days, respectively. We followed the IUPAC guidelines to evaluate the LOD and selectivity. Acceptable method validation parameters confirm the validity of the developed potentiometric method, Table 3. The dynamic response time plot, Supplementary Figure S5, shows that the sensor expressed a short equilibration time, after which the response remained stable with minimal potential drift over the concentration range 1.1 × 10^–6^–1.0 × 10^–6^ M, with an average response time of 6.5 s. The fast response is due to the enhanced exchange kinetics of HCQ at the membrane solution interface, whereas the stable HCQ-BCD inclusion complex maintained a stable sensor response and minimized potential drift. The sensor proved a steady slope ± 1 mV for 6 successive weeks; afterward, the slope declined due to the leakage of the membrane components in the storage solution.

### Comparison to the reported HPLC method for the determination of HCQ

The developed sensor showed better analytical merits for HCQ determination over the reported HPLC method^38^. The sensor is more sensitive and selective, suitable for in-field analysis, and effective in cost and time. The potentiometric sensor possesses higher greenness potentials than the reported HPLC in terms of lower chemicals, solvents, energy consumption, and minimal waste production. Besides, the results were statistically comparable to those obtained by the reported HPLC method^38^. The student t-test and F-test values revealed no significant difference concerning accuracy and precision, Supplementary Table S6.

## Conclusion

This work produced a quality-by-design, simple, and efficient potentiometric sensor for the at-line determination of HCQ in the presence of its toxic impurities. We adopted a comprehensive and timely QBD approach to evaluate the effect of the sensor composition, namely ion exchanger, plasticizer, ionophore, and their interactions on the sensor performance. The desired performance was observed using tetraphenyl borate, dibutyl phthalate, and β-cyclodextrin in the polyvinyl chloride matrix. The optimized sensor proved a high correlation coefficient and a short response time of 6.5 s within a broad pH range of 2.5–8.5. The selectivity of the optimized sensor allowed the direct determination of HCQ in the presence of its key starting materials and toxic synthesis impurities, DCQ, and HND. In addition to being a purity-check method, the proposed sensor offers a cheap, timely, affordable, user and environmentally friendly industrial tool to monitor HCQ during synthesis with minimum resource consumption and waste production.

### Supplementary Information

Supplementary Information.

## Data Availability

The datasets employed and/or scrutinized during this study can be obtained from the corresponding author upon a duly substantiated request.
