# Supplementary material for: Quality-by-design ecofriendly potentiometric sensor for rapid monitoring of hydroxychloroquine purity in the presence of toxic impurities
Source: Sci Rep. 2024 Mar 22;14:6869. doi: 10.1038/s41598-024-53456-8 (PMC10960021; doi:10.1038/s41598-024-53456-8)
Supplement: Supplementary file 1 — Supplementary Information. [file 41598_2024_53456_MOESM1_ESM.docx]

**Supplementary Materials**


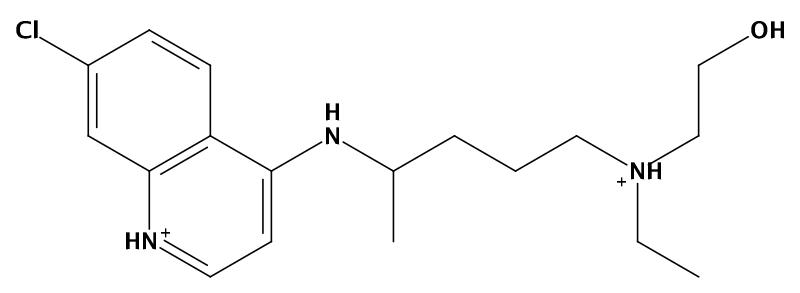

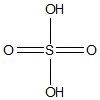

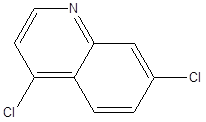

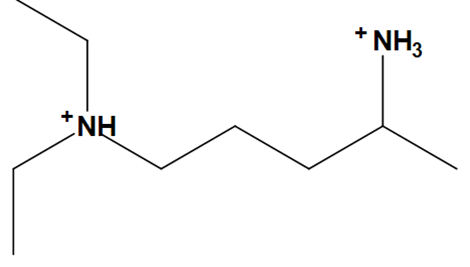


(a)

(c)

(b)

**Supplementary Figure S1.** Chemical structures of (a) hydroxychloroquine sulfate, (b) 4,7-Dichloroquinoline, and (c) hydroxynovaldiamine.

**
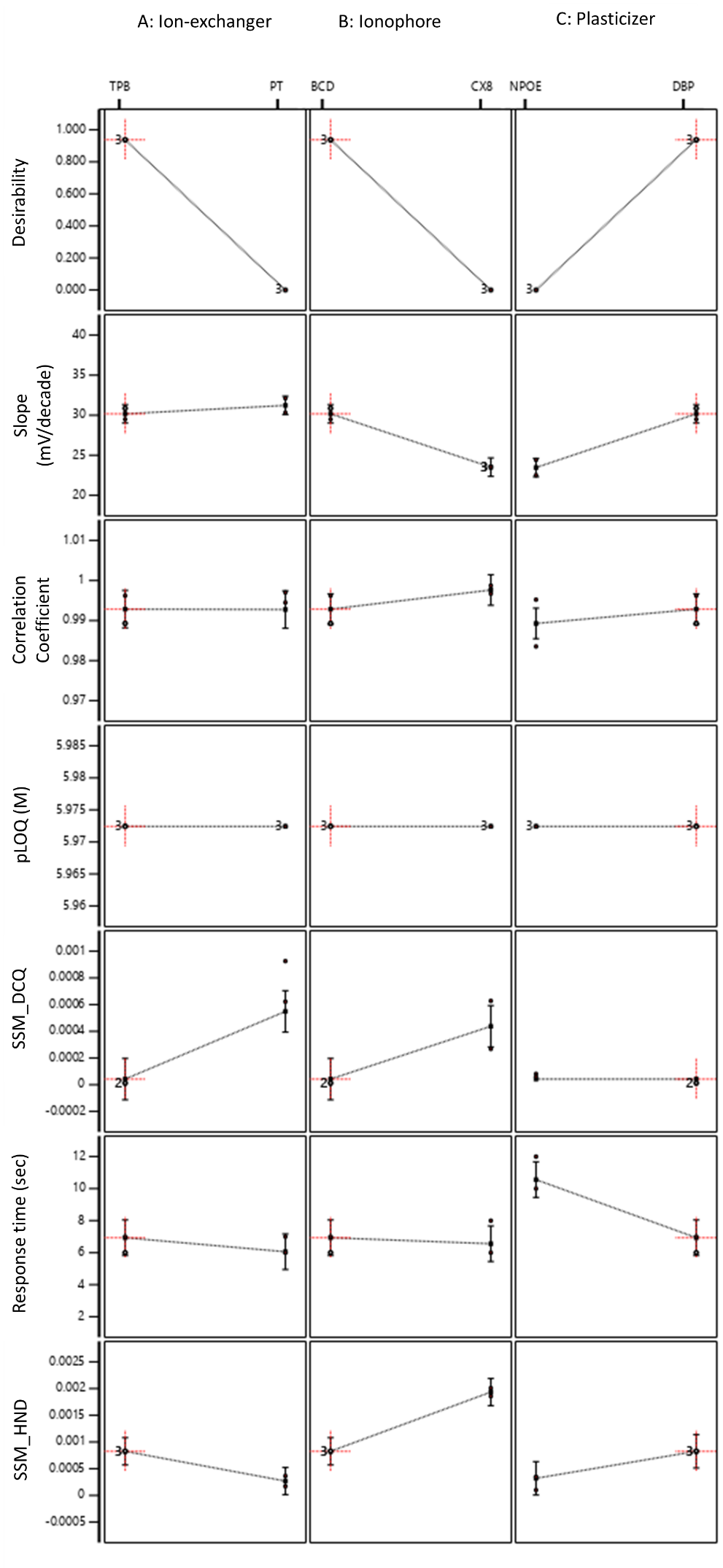
Supplementary Figure S2.** Desirability function and main effects for the ion-exchanger, ionophore, and plasticizer on each studied response.

**Supplementary Figure S3.** Effect of pH on the measured potential of 1.00×10^-2^ and 1.00×10^-3^ M HCQ solutions using the developed sensor.

**Supplementary Table S4.** Potentiometric selectivity coefficients $K_{HCQ, Int}^{pot}$values of the optimized sensor for different interfering cations.

| **Interfering compound** | $\boldsymbol{K}_{\boldsymbol{HCQ, Int}}^{\boldsymbol{pot}}$ |
| --- | --- |
| 4,7-Dichloroquinoline | 3.94 ×10^-5^ |
| Hydroxynovaldiamine | 8.40 ×10^-4^ |
| K^+^ | 3.55 ×10^-5^ |
| Na^+^ | 5.17 ×10^-5^ |
| Ca^+2^ | 3.55 ×10^-5^ |
| Pb^+2^ | 2.62 ×10^-5^ |
| Cd^+2^ | 2.26 ×10^-5^ |
| Ni^+2^ | 1.44 ×10^-5^ |
| Mg^+2^ | 1.55 ×10^-5^ |
| Mn^+2^ | 3.05 ×10^-5^ |
| Fe^+3^ | 2.43 ×10^-5^ |
| Cu^+2^ | 3.55 ×10^-5^ |
| NH_4_^+^ | 1.94 ×10^-5^ |
| Co^+2^ | 3.05 ×10^-5^ |


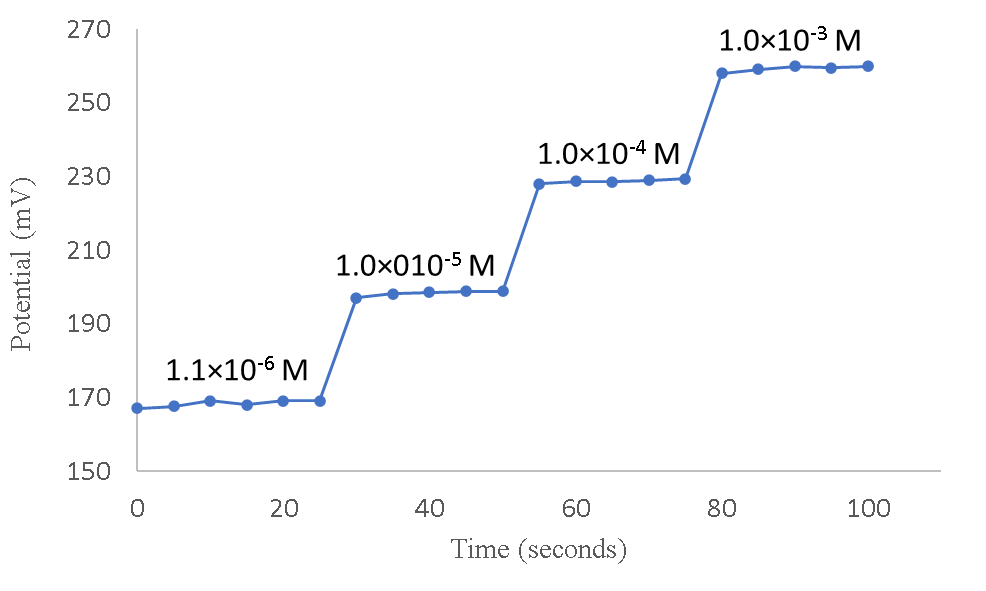


**Summplentary Figure S5.** The Dynamic potentiometric response of the optimized sensor for different hydroxychloroquine concentrations.

**Supplementary Table S6.** Statistical comparison of the proposed potentiometric and reported HPLC methods for determining hydroxychloroquine in Plaquenil^®^ tablets.

| **Parameters** | **Proposed potentiometric method** | **Reported HPLC method** ^37^** |
| --- | --- | --- |
| **Mean %** | 100.34 | 100.38 |
| **SD** | ±1.16 | ±0.72 |
| **n** | 6 | 6 |
| **Student *t*-test** | 0.077 (2.228)***** | -------------- |
| ***F*- value** | 2.589 (5.050)***** | --------------- |

- Parentheses' figures represent the corresponding t and F tabulated at P= 0.05.

****** The separation was conducted on C18 (250 × 4.6 mm) HPLC column with acetonitrile: methanol: KH_2_PO_4_ (10:10:80) mixture containing 0.01% triethylamine.
